# Fluorescence-guided minimally-invasive resection of abdominal paragangliomas using indocyanine green

**DOI:** 10.1038/s41598-024-54718-1

**Published:** 2024-02-17

**Authors:** M. A. van Dam, A. S. L. P. Crobach, B. Boekestijn, E. P. M. Corssmit, B. A. Bonsing, A. L. Vahrmeijer, J. S. D. Mieog

**Affiliations:** 1 Department of Surgery, Leiden University Medical Center, Leiden, The Netherlands; 2 Department of Pathology, Leiden University Medical Center, Leiden, The Netherlands; 3 Department of Radiology, Leiden University Medical Center, Leiden, The Netherlands; 4 Division of Endocrinology, Department of Internal Medicine, Leiden University Medical Center, Leiden, The Netherlands

**Keywords:** Endocrine system and metabolic diseases, Endocrine system

## Abstract

This retrospective study explores the utility of near-infrared (NIR) fluorescence imaging with indocyanine green (ICG) in enhancing the intraoperative identification and guidance for the resection of abdominal paragangliomas. They can be challenging to detect during minimally invasive surgery, due to their anatomical location, varying size and similar appearance in regard to their surrounding tissue. Patients with suspected abdominal paragangliomas planned for a minimally-invasive resection were included. As part of standard of care they received single intravenous dose of 5 mg ICG after abdominal exploration. NIR fluorescence imaging of the anatomical region of the suspected lesion was performed immediately following intravenous administration, to assess fluorescence signals, intraoperative identification, and histopathological correlation. Out of five resected suspicious lesions, four were imaged with NIR fluorescence, pathology confirming four as paragangliomas, the latter turned out to be an adrenal adenoma. NIR fluorescence identified all four lesions, surpassing the limitations of white-light visualization. Homogeneous fluorescence signals appeared 30–60 s post-ICG administration, which lasted up to 30 min. The study demonstrates the feasibility and potential clinical value of fluorescence-guided minimally-invasive resections of abdominal paragangliomas using a single intravenous ICG dose. These findings support the scientific basis for routine use of ICG-fluorescence-guided surgery in challenging anatomical cases, providing valuable assistance in lesion detection and resection.

## Introduction

Paragangliomas are a rare type of neuroendocrine tumors, with an estimated incidence of 1/100.000/year peaking between 30 and 50 years old^1^. Paragangliomas, originating from chromaffin cells, develop alongside arteries or (para)sympathetic nerve ganglia in various body regions including the head, neck, abdomen, or pelvis^2–4^. Abdominal paragangliomas typically arise from retroperitoneal paraganglia, symmetrically positioned along the abdominal aorta. Common locations include the infrarenal area near the origin of the inferior mesenteric artery or adjacent to the organs of Zuckerkandl^5,6^. Paraganglionic tissue is well vascularized due to their catecholamines secreting cells embedded around multiple fenestrated capillaries^6^. Most sympathetic paragangliomas produce an excessive amount of catecholamines and their metabolites (metanephrines), with symptoms related to catecholamine excess, such as hypertension, cardiac arrythmias and headaches^4^. However, up to 20–30% of patients remain asymptomatic, resulting in incidental discovery of the tumor during routine radiological imaging (incidentaloma), typically with CE-CT or MRI^6,7^. The diagnostic work-up of patients presenting with symptoms, associated with a catecholamine-excess, consists of a biochemical evaluation of plasma or urine samples to identify increased urinary fractionated or plasma-free metanephrines levels^8^. Biochemical evaluation is accompanied by a CE-CT or MRI to localize potential foci and perform general staging. Furthermore, functional imaging can be utilized to distinguish between functional and nonfunctional paragangliomas. This includes ^123^I-Meta-iodobenzylguanidine (MIBG) scintigraphy, Positron-Emission Tomography (PET)-CT using radiolabeled nucleotides such as ^18^F-Fluorodeoxyglucose (FDG), as well as somatostatin receptor tracers like ^68^ Ga-dotatate and ^111^In-Pentetreotide (octreotide)^6^.

Paragangliomas are known to be associated with germline mutations, up to now, 21 germline mutations have been discovered^9^. In approximately 30–40% of the cases a germline mutation is discovered during standard of care diagnostic work-up^9,10^. While most paragangliomas are benign, about 15–20% become malignant, metastasizing to osseous and hepatic sites, with limited and rarely curative treatment options^10–13^. Therefore, early identification and complete surgical removal is usually curative and plays a crucial role in achieving a favorable prognosis^14^. Treatment is in baseline indicated for all patients diagnosed with paraganglioma, especially in case of patients with catecholamine excess related symptoms. Curative treatment primarily consists of complete resection and, in case of functional lesions, preoperative alpha-blockade to mitigate potential catecholamine release effects during surgery^1,15^. Abdominal paragangliomas can be resected using either an open or minimally-invasive approach, with a preference for the minimally-invasive method if it is deemed safe and feasible^1^. However, successful identification and complete removal of one or more suspected lesions can be challenging, primarily due to their close-relation with major abdominal vasculature, localization in the retroperitoneal compartment or varying number and size of the lesions^16^. This complexity could be further increased during minimally-invasive procedures, which are characterized by reduced tactile feedback. Since tactile perception helps surgeons in tissue distinction and maneuvers, this could pose challenges for surgeons to adequately demarcate the lesion, decide on the resection plane, and safely navigate within the retroperitoneal cavity during the procedure^17^. Near-infrared (NIR) fluorescence imaging, also known as fluorescence-guided surgery (FGS), is an increasingly applied intraoperative technique that provides surgeons with real-time visual feedback of tissues of interest and crucial anatomical structures during surgical procedures^18^. FGS uses fluorescent tracers that absorb and emit near-infrared light (λ = 700–1300 nm), detectable by a NIR fluorescence imaging system^18,19^. Intraoperative identification of solid tumors and peritumoral vasculature using NIR fluorescence imaging has been demonstrated across various tumor types, including neuroendocrine tumors in the adrenal glands, (para)thyroid glands, liver, and pancreas. Existing literature primarily focused on fluorescence-guided adrenalectomies for various types of adrenal neoplasms, including pheochromocytomas^20–22^. However, the utilization of FGS specifically for (abdominal) paragangliomas has remained largely unexplored. Tummers et al*.* describe in their case report the successful identification of an abdominal paraganglioma in a 19-year-old patient using the fluorescent tracer, Methylene Blue (MB)^23^. Nevertheless, this tracer presents certain substance-related limitations when compared to the use of indocyanine green (ICG), such as compatibility issues with clinically available imaging systems due to its near-infrared (NIR) wavelength of 700 nm and the associated increased risk of autofluorescence. ICG is the most frequently used and FDA-approved fluorescent contrast agent for FGS^24^. It is a water-soluble dye which binds to plasma lipoproteins (albumin) and has a short half-life time of 3 to 4 min. ICG distributes rapidly via the bloodstream and is exclusively excreted through the hepatobiliary system, appearing unconjugated in the bile^25,26^. One of the notable advantages of ICG is its compatibility with clinically available fluorescent imaging systems with an 800 nm channel. The mechanism for identifying solid tumors, including paragangliomas, using ICG-FGS is hypothesized to rely on the biological mechanism of increased accumulation and prolonged retention of small molecules (> 40 kDa), such as ICG, within the altered vascular microenvironment. This environment is characterized by a defective endothelial cell lining, widely fenestrated microcirculation, and upregulated secretion of vascular permeability factors, resulting in enhanced permeability and accumulation of ICG in solid tumors, along with prolonged retention due to the physiological properties of the dyes^27^. This mechanism is referred to as the Enhanced Permeability and Retention (EPR) effect, initially described by Matsumura and Maeda in 1986^28^. Building on this concept and the findings of previous series evaluating ICG-FGS in neuroendocrine tumors, we investigated the potential to identify paragangliomas during minimally-invasive surgery using ICG-guided NIR fluorescence imaging in a retrospective cohort study.

## Methods

### Registration and ethics

This cohort study was registered in the clinicaltrials.gov database (NCT06155734), and adherence to the STROBE guidelines for reporting observational studies was ensured^24^. This retrospective cohort study received approval from the scientific committee of the Leiden University Medical Center (LUMC) in the Netherlands. Formal ethical approval was waived due to the retrospective nature of the study and considered not applicable according to the Medical Research Involving Human Subjects Act and the World Medical Association (WMA) Declaration of Helsinki. All patients received a single intravenous dose of ICG as part of standard surgical care, and clinical and imaging data were retrospectively retrieved from the hospital’s electronic patient files after obtaining formal informed consent.

### Design and patients

This study was conducted at a single academic institution, the Leiden University Medical Center (LUMC), in the Netherlands. Patients who were diagnosed and underwent minimally invasive resection using NIR fluorescence imaging for an abdominal paraganglioma between January 2021 and November 2023 were included after informed consent was obtained. All patients were preoperatively informed by the treating surgeon about the standard of care use of ICG for intraoperative NIR fluorescence imaging. Patients received standard of care treatment and follow-up. All relevant clinical, surgical, pathological, and imaging data were retrieved from the local electronic patient files or from the local tissue repository. Two academic surgeons (JSD, BA), both with more than 10 years of experience in minimally invasive gastrointestinal, hepatopancreaticobiliary and oncological surgery, as well as experience in FGS, performed all procedures.

### Indocyanine green (ICG) and near-infrared (NIR) fluorescence imaging

**Figure 1:**
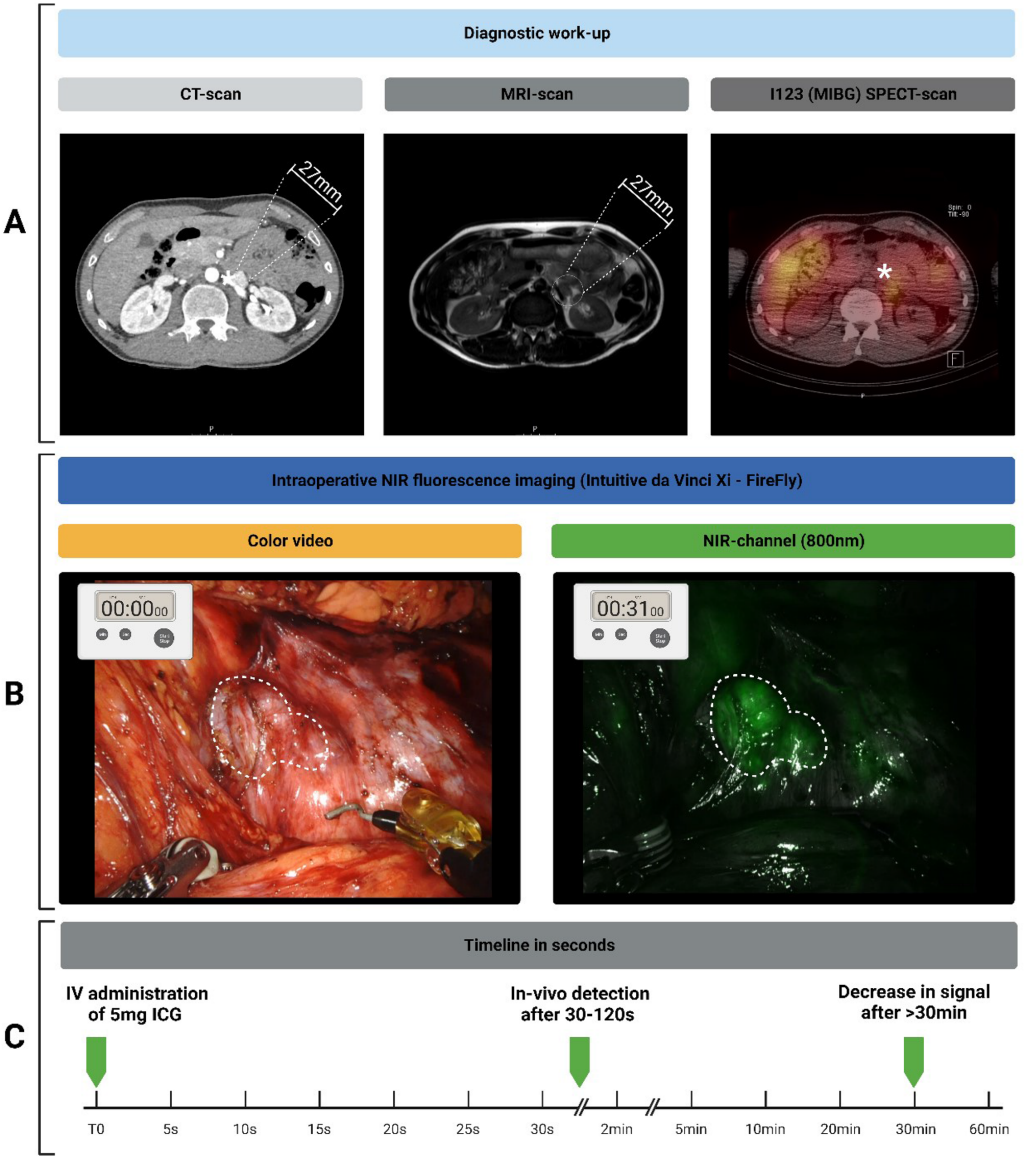
Multimodal workflow of perioperative diagnostics of subject 1. Detailed overview of subject 1, diagnosed with a 27 mm lesion on the cranial side of the left renal hilus, just above the renal artery. Suspected for paraganglioma, based on abdominal CT, MRI and additional I^123^ * Corresponding location on different imaging modalities (**A**). Surgical robot-assisted resection and NIR fluorescence imaging was performed using the Intuitive da Vinci Xi surgical robot in FireFly-mode, t = 31 s after IV administration of Indocyanine Green (ICG) homogenous fluorescence signal was detected using the NIR-channel (**B**). Administration-Imaging timeline for NIR-fluorescence imaging of abdominal paragangliomas (**C**). Created with biorender.com. *CT* contrast-enhanced computerized tomography; *ICG* Indocyanine Green; *IV* Intravenous; *NA* not applicable; *NIR* Near-infrared Fluorescence; *MIBG* Meta-iodobenzylguanidine; *MRI* Magnetic Resonance Imaging.

Indocyanine green (ICG; Verdye, Diagnostic Green Ltd, Ireland) was prepared according to the manufacturer's prescriptions by dissolving a vial of 25 mg in 10 ml of sterile water for injection, yielding a 2.5 mg/ml (3.2 mM) stock solution. After visual inspection of the abdomen, surgical exploration, and identification of the suspected anatomical region of interest, 5 mg (2 mL) ICG was intravenously administered as a single bolus, and the line was flushed with 10 ml of NaCl (0.9%), referenced to the method described for perfusion assessment in gastrointestinal surgery^29^. During laparoscopic resection, the NIR channel and LED light source of the Image 1S RUBINA laparoscopic imaging system (KARL STORZ SE & Co. KG—Tuttlingen, Germany) were used. During robot-assisted resection, the da Vinci Xi (Intuitive, Sunnyvale, California, United States of America) robot-assisted surgical system, with the FireFly operating mode, was used. Static and moving images of the procedures were recorded during the interventions as part of standard of care procedures (Fig. 1).

### Analysis and statistics

All static and moving images of the included patients were retrieved to analyze image quality, fluorescence signal, administration-to-identification time window, and correlation to histopathological outcome. Descriptive statistics were used for patient and clinical characteristics as well as the primary outcome. Descriptive statistics were summarized using the mean and standard deviation or median and range.

## Results

### Study cohort and surgical outcome

A total of four patients were enrolled in this study, of which three patients underwent a robot-assisted resection and one patient a laparoscopic resection (Table 1). Median age was 40 years and two were female. In total, five lesions were suspected for abdominal paragangliomas based with the longest lesion diameter ranging from 15 to 48 mm on preoperative imaging. The median operating time ranged from 148 to 182 min. No complications occurred in the 30-day period following surgery and no (serious) adverse events related to the intravenous administration of ICG occurred. All five identified suspected lesions were resected with radical (R0) surgical margins. Four out of five resected lesions were histopathologically classified as a paraganglioma. One lesion, more suspect for a pheochromocytoma based on its anatomical location, was classified as an adenoma of the left adrenal gland. All patients diagnosed with a paraganglioma on definitive diagnosis had a related SDHD germline mutation. Standard of care follow-up up until six months after resection showed no signs of (locoregional) recurrence or persisting elevated metanephrine levels related to potential foci left in situ. Standard follow-up for patients with a definitive diagnosis of paraganglioma consisted of abdominal CE-CT scans or whole-body MRI scans, following a dedicated paraganglioma screening protocol. In the case of subject #3, diagnosed with adenoma, further routine radiological follow-up was not indicated.

**Table 1:** Demographics of study cohort.

	Age	Gender	Anatomical region	Surgical approach	Dose	WLI identification	FLI identification	Size of lesion based on CT-scan	Pathological diagnosis	Residual tumor (R) classification	Genetic mutation
Subject 1	38	M	Left renal hilum	Robot-assisted resection	5 mg i.v	+	+	27 mm	Paraganglioma	R0	SDHD
Subject 2	41	F	Right adrenal gland	Robot-assisted resection—enucleation	5 mg i.v	+	NA	18 mm	Paraganglioma	R0	SDHD
			Uncinate process of the pancreas			- * Ultrasound	+	22 mm	Paraganglioma	R0	
Subject 3	39	F	Left adrenal gland	Robot-assisted resection	5 mg i.v	+	+	48 mm	Adenoma	R0	–
Subject 4	67	M	Ileocolic mesentery	Laparoscopic resection	5 mg i.v	–	+	14 mm	Paraganglioma	R0	SDHD

Demographics. Overview of the study cohort, in total four subjects have been included * The lesion in the uncinate process of subject 2 was not identified with WLI, intraoperative ultrasound using the ultrasound probe in the Da Vinci Xi surgical robot was able to localize the lesion. Residual tumor is classified according to the Union for International Cancer Control (UICC) Residual tumor (R) classification: R0, Radical resection; R1, Microscopic residual tumor; R2, Macroscopic residual tumor^30^.

*FLI* Fluorescence imaging; *i.v*. Intravenous; *NA* not applicable; *R0* Radical resection; *R1* Microscopic residual tumor; *R2* Macroscopic residual tumor; *SDHD* succinate dehydrogenase subunit-D; *WLI* White-light imaging.

### Results of intraoperative fluorescence imaging

**Figure 2:**
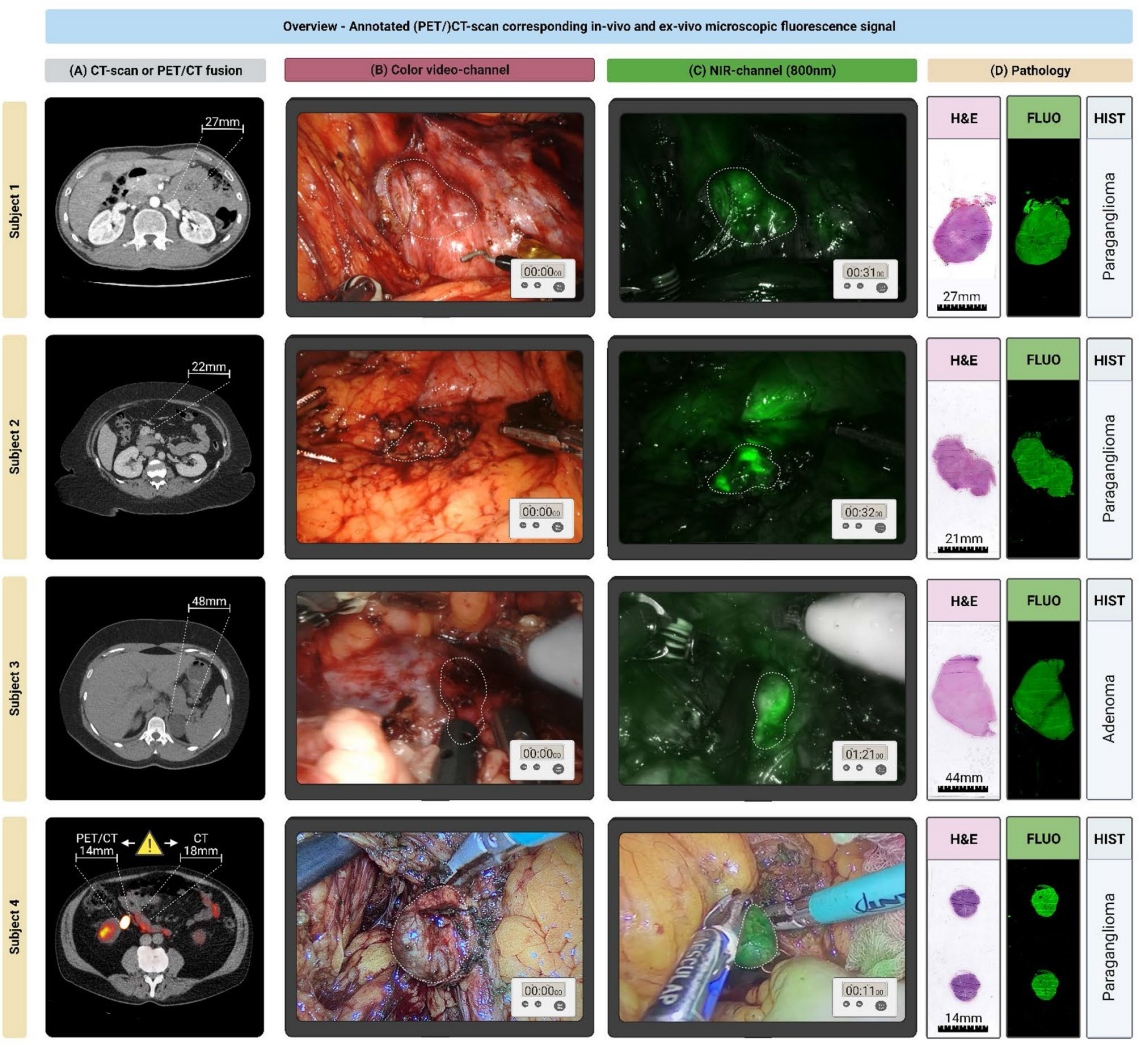
Multimodal overview of included cases, referenced to histopathology. Detailed overview of subject 1–4 with preoperative (PET)/CT-scan used during the diagnostic work-up (**A**), Color-video image of surgical field (**B**), the in-vivo identification in the suspected tumor during minimally-invasive procedures (**C**), Hematoxylin and eosin (HE) stained resection slides and corresponding ex-vivo NIR fluorescence scans of blank histology slides (**C**) [LI-COR Odyssey CLx, Lincoln, NE, U.S.A.]. Subject 4 (Panel A): Based on the preoperative work-up there was a discrepancy in the suspected lesion location and diameter based on CT and ^68^ Ga-Octreotide PET/CT-scan, in-vivo the lesion corresponding with the ^68^ Ga-Octreotide PET/CT-scan was identified. Subject 4 (Panel D): Microscopic slide with two 4um tissue sections of the same lesion. (Subject 1–3: da Vinci Xi [Intuitive, Sunnyvale, California, United States of America] robot-assisted surgical system, in FireFly-operating mode; Subject 4: Image 1S RUBINA laparoscopic imaging system [KARL STORZ SE & Co. KG – Tuttlingen, Germany] in overlay-operating mode). Created with biorender.com. *CT* contrast-enhanced computerized tomography; ^68^ Ga, 68-Galium; *HE* Hematoxylin and eosin; *ICG* Indocyanine Green; *MRI* Magnetic Resonance Imaging; *NIR* Near-infrared Fluorescence.

Intraoperative identification of the suspected tumor using standardized white-light visualization (WLI) was successful in 3 out of 5 lesions (Table 1). Near-infrared (NIR) fluorescence imaging was performed for the identification of 4 out of 5 lesions, as one subject (#2) had two suspect lesions, and ICG-FGS was only performed to identify the lesion in the uncinate process of the pancreas. In all four imaged cases, the suspect paragangliomas were identified and successfully resected using ICG-FGS (Fig. 2). Two lesions were identified solely by using NIR fluorescence imaging, as they were not identified with WLI. One of these lesions, located in the uncinate process of the pancreas, was also visualized using intraoperative ultrasound. Subject #3, who was preoperatively diagnosed with an incidentaloma adjacent to or originating from the left adrenal gland, was ultimately diagnosed with an adenoma instead of a paraganglioma based on definitive histopathology. Nonetheless, the adenoma showed similar homogeneous fluorescence signals intraoperatively to the other cases with a definitive diagnosis of paraganglioma. Subject #4 previously underwent an unsuccessful robot-assisted procedure for resection of a suspected paraganglioma located medioventral to the right kidney in close proximity to the ileocolic artery. Preoperative localization of the suspected lesion was based on a combination of CE-CT, MRI, and ^68^ Ga-dotatate PET/CT scan. However, during this procedure, the suspected mass could not be clearly identified using standard WLI. In the region of the ileocolic artery, an irregular lymphoid structure was identified, resected, and sent in for histopathological analysis. Histopathological analysis showed a normal lymph node. During a second laparoscopic procedure, ICG-FGS was performed. During this procedure, the region of the ileocolic artery was explored, where an irregular spherical lesion was identified with an intense demarcating fluorescence signal. After confirmation of the paraganglioma diagnosis with freeze-biopsy, the mass was successfully resected.

### Qualitative analysis of near-infrared signals

In general, the inflow of fluorescence signal into the suspected paraganglioma tissue was observed within 30–60 s, with a peak signal sufficient to identify the suspected tumor occurring around a mean of 39 (SD ± 26) seconds after intravenous administration (Supplementary video 1). Homogeneous fluorescence signals over time were observed for all imaged lesions ranging from 1 to 30 min after ICG administration, based on the available imaging time points. A wash-out of ICG, indicated by a decreasing signal, was typically observed from tumor tissue after more than 30 min.

## Discussion

In this cohort study, we demonstrate the feasibility and added value of intraoperative identification of paragangliomas with ICG-FGS. Using a single intravenously administered bolus of ICG (5 mg) a homogeneous fluorescence signal was observed in all lesions within 1–2 min after intravenous administration. In this series of four patients, NIR fluorescence imaging proved to be beneficial in all cases. It successfully identified the preoperatively identified mass in two cases where conventional white light inspection failed, and it also confirmed the lesion's location in the remaining two cases. Our findings suggest that the use of ICG-FGS should be considered during the resection of abdominal paragangliomas, particularly in cases where the lesion is situated in a difficult anatomical location.

NIR fluorescence imaging using ICG as a surgical tool for identification, navigation and decision-making during resection has been previously described in more detail for several indications, including benign disease as well as malignant tumors; for example for identification of parathyroid adenomas^31–34^; primary and secondary liver tumors^35–40^ and peritoneal metastases from colorectal or ovarian origin^41–45^. However, distinctions can be made within these applications based on the underlying mechanisms of ICG inflow, accumulation, retention, and clearance. For example, in primary and secondary liver tumors, ICG needs to be administered days before detection, as opposed to hours to minutes. This is due to its underlying mechanism involving hepatic metabolism of ICG and its subsequent accumulation within hepatocellular carcinomas or the surrounding hepatic parenchyma of liver metastases, due to an impaired parenchymal metabolic function^46^. Whereas, for identification of parathyroid adenomas, ICG is administered intra-operatively 1–2 min before supposed signal detection^32^. Matson et al*.* demonstrated that relying on the underlying mechanism of vascular flush and retention induced by enhanced permeability of the vessels, ICG exhibited rapid inflow and accumulation in adenomas, resulting in the detection of the fluorescence signal within minutes after administration^32^. Our observations are consistent with previous research findings, revealing that ICG demonstrated a comparable fluorescence inflow and retention pattern in paragangliomas. This pattern was detectable within 1–2 min after administration. This rapid vascular inflow is similar to that used in ICG-guided assessment of intestinal perfusion during abdominal procedures, which is one of the most commonly known indications for the use of ICG-FGS^47–50^. Tummers et al. showed similar results in their case-report, which explored the intraoperative detection of paragangliomas using methylene blue (MB)^23^. They observed a similar rapid inflow of MB as perfusion effect, in regard to the retention effect with a rapid decrease in fluorescence signal over time. In the context of pheochromocytomas, the existing literature presents varied findings regarding fluorescence status. Berber et al. recently demonstrated feasibility of minimally-invasive identification of suspected pheochromocytomas using ICG fluorescence imaging. They observed a similar inflow and retention pattern, but noted that only half of the adrenal lesions exhibited any fluorescence signal. Through a multivariate analysis, they tested their hypothesis of a correlation between the lesion's fluorescence status and its size, finding a positive (fluorescent) correlation in lesions with a minimal diameter of 32 mm^22^.

The retrospective nature of this small cohort study results in some known limitations. Since the data were collected retrospectively from the electronic patient files after informed consent, images and videos taken during the procedures were not made according to a standardized study protocol, i.e., standardized distance from organ to camera, angle between camera and target tissue, etc. Therefore detailed quantification of the in, and outflow of fluorescence signals was not possible. Furthermore, because patients who received 5 mg of ICG preoperatively as standard of care, we were not able to conduct dose ranging and dose-timing optimization for the single bolus administration of ICG. It is possible that comparable results could be achieved with a reduced single dose of ICG. Moreover, an analysis of operative duration between fluorescence-guided surgery (FGS) resections employing ICG and non-FGS resections was not conducted to evaluate its impact on the overall surgical time. Nevertheless, the influence on total surgical time could be mitigated by considering the two cases wherein the suspected paraganglioma was solely identified using ICG; one could imagine that the limited additional time used for ICG-FGS was relatively compensated by the added time for identification when solely WLI was performed. One of the suspected paragangliomas, exhibiting a similar fluorescence pattern, was eventually confirmed to be an adenoma originating from the adrenal gland. Therefore, the fluorescence pattern itself is nonspecific for paragangliomas, and one could argue it as a false-positive finding. For future research, these aspects should be considered and incorporated in prospective series evaluating ICG-FGS. Nevertheless, the primary aim of the study was to present an insight into the use of a ICG-FGS using a single intravenous dose of 5 mg for real-time intraoperative identification of suspected paragangliomas.

Surgical resection is the recommended treatment for paragangliomas, emphasizing the necessity of precise tumor identification for achieving thorough and minimally-invasive resections. The trend towards minimally-invasive laparoscopic- and robotic-assisted surgery underscores the increasing significance of achieving optimal intraoperative tumor identification and demarcation. We believe our findings add the growing body of literature examining the potential benefits of intraoperative ICG fluorescence guidance in minimally-invasive resections. Specifically, ICG-FGS could be a valuable tool, enabling surgeons to receive real-time assistance during the minimally-invasive resection of abdominal paragangliomas.

### Supplementary Information

Supplementary Video 1.

## Data Availability

The data that support the findings of this study are available on request from the corresponding author.
